# Prevalence of bifidity of the seventh cervical vertebral spinous process in southwestern Nigeria: a computed tomography based study

**DOI:** 10.1038/s41598-024-51998-5

**Published:** 2024-03-31

**Authors:** Babatunde Oluwaseun Ibitoye, Olatunde Wasiu Oladipupo, Fransisca Omolara Ibitoye, Olumide Akadiri, Olajumoke Fatima Bello

**Affiliations:** 1 Department of Anatomy, Ekiti State University, Ado Ekiti, Nigeria; 2 Department of Radiology, James Paget, University Hospital, Great Yarmouth, Norfolk, UK; 3 Department of Science Laboratory Technology, Rufus Giwa Polytechnic, Owo, Ondo State Nigeria; 4 Department of Obstetrics and Gynecology, University of Medical Science Teaching Hospital, Akure, Ondo State Nigeria

**Keywords:** Biological techniques, Biotechnology, Evolution, Structural biology, Anatomy

## Abstract

Palpation of the seventh cervical vertebra (C7) is an important landmark for counting vertebrae for vertebral spine surgical instrumentation. However, studies have shown that the spinous process of C7 displays an anatomical deviation among individuals, which may mislead a surgeon who is not aware of this, and there have been no such studies among southwest Nigerians. The present study aimed to examine the incidence of bifidity in the C7 spinous process and their variation among 48 subjects with the aid of a three-dimensional (3D) computed tomography. The 48 subjects who had undergone cervical spine computed tomography studies comprised the pooled data of ages between 12 and 55 years of black race in southwest Nigeria from the radiology department of the Trauma and Surgical Centre, Ondo State, Nigeria. A series of multivariate and discriminant statistical tests were performed on the measurement data to determine the occurrence of bifid spinous processes at C7 in southwestern Nigeria. The results show about 10% bifidity in the C-7 vertebra and no bifidity in the first cervical vertebra and the highest rate in the C-6 vertebra in the study population. The incidence in this study is significantly higher than findings in previous works. Thus, there is a need for clinicians to pay more attention to this variation when using C7 as a landmark especially in the studied population.

## Introduction

The seventh cervical spinous process (C7), also known as vertebra prominent, is an atypical, non-bifid-tipped process that can be felt posteriorly at the base of the neck, unlike typical cervical vertebrae such as C2–C6, which have the feature of a variable degree of bifidity. The average person in the health sector who has had to pass through anatomy class is mostly aware of this^1^. In the early twentieth century, Shore reported on an extensive anatomical investigation of the spinous processes of the cervical vertebrae^2^. If a spine is divided into two at the tip, it is referred to as a bifid spine^3^. The third to sixth cervical vertebrae have what is known as a bifid spine^4^.

However, the incidence of C7 bifidity has been seen to be low the in studied white population where only 0.3% prevalence was found^5^. The prevalence of cervical spine bifidity has been observed to vary from race to race, in a study of 359 Americans of African (black) and European (white) ancestry, bifidity was shown to be substantially more common in the white South African sample than the black group, which may indicate a demographic difference^6,7^. It was reported by Shore (1931) that there is a higher incidence of bifidity in foetuses of both black and European descent, but a higher prevalence of monofid in the black could explain ossification at adulthood, which is said to be more accentuated in blacks due to stress on the cervical spine induced by the carrying of heavy loads on the head, as opined by Anas et al. (2010)^2,4^ Shore (1931) was of contrary opinion that bifidity could have evolved from monofid cervical spine. In a case report, Das et al. 2005, described the duplicated spinous process of the C7 vertebra and that the appearance of bifid spines, which are divided in half at the tip, has been attributed to the growth of two ossification centers^3,8^.Since its incidence varies among different races, this makes spinal bifidity an anthropological indicator for forensic usage^9^.

The cervical spine C2 to C6 is considered to be an unreliable landmark in the posterior cervical spine approach due to the degree of variability in the incidence of the bifurcation in their cervical spinous processes^10^. This makes the seventh cervical spinous process crucial for anaesthesiologists to count levels when inserting epidural catheters^11^. Forensic and anthropological research as well as daily clinical practice by radiologists, neurologists, and orthopaedists are all impacted by the existence of a non-bifid c7 spinous process^12^. The absence of the vertebral artery at C7, which could have been harmed during the treatment, has made this approach for transpedicular fixation thought to be safer^13^. The reliability of the cervical spinous process bifurcation as a landmark in the posterior cervical spine approach has been diminished as a result of Moro et 2007, a study of the frequency of bifurcation of the spinous process tip in 50 specimens, C2–C4 were all bifid, but 47; C5, C6, and 21; C7, were bifid^14^. A study by Cho et al. in 2012, found that 0.3% of the time a genuinely bifid C7 spinous process occurred^11^. Due to the rarity of this occurrence, it is crucial to be aware of it in order to avoid performing the incorrect degree of instrumentation and causing misinterpretation in radiological examinations as a result of incorrectly counting the cervical spines in a skiagram.

The cervical spinous processes are subjected to variations in regards to the length, types, and mode of bifurcations and deviation from the median plane^15^. These abnormalities can be asymptomatic or detected incidentally, or mimic traumatic lesions and may cause recurrent episodes of pain affecting the quality of life^16^. Congenital variations of the cervical spine can be associated with other abnormalities such as VACTERL (vertebral anomalies, anal atresia, cardiac malformations, tracheoesophageal fistula, renal anomalies, limb abnormalities) association or clinical syndromes such as Klippel-Feil and Morquio or dystrophic dwarfism, spondyloepiphyseal dysplasia, and osteogenesis imperfect^17^.

This work is being taken into consideration since it might help raise awareness of this intriguing anomaly, which could be crucial for radiologists, orthopaedic surgeons, and neurologists. Spinous processes in the cervical vertebrae are typically bifid. Yet, this characteristic might differ between subjects and even within a single vertebra of the same person. Such diversity has not yet been the focus of any in-depth investigations, despite the fact that it can be significant in forensic medicine, anthropological studies, and archaeological studies. Also, no study of this kind has been done among the black population. It is also worth noting that a 3D reconstruction of the image gotten from a CT scan will be used as opposed to an earlier study by Cho et al. (2012), which was 2D and subject to a greater degree of error. The aim the aimed of the study is to examine the incidence of bifidity in the C7 spinous process and their variation among 48 subjects with the aid of a three-dimensional (3D) computed tomography.

## Materials and methods

This is a retrospective study carried out at the radiology department of the Trauma and Surgical Center, Ondo State. The study consisted of 48 subjects who had undergone cervical spine studies between January 2013 and January 2017, accessed from the picture archiving and communications system (PACS) database of the department after Institutional Review Board approval by Ethical and research committee of Anatomy department, Ekiti State University, Ado Ekiti, Ekiti state, with protocol number ERCANA/2023/02/001. The research was carried out in accordance with relevant guidelines and regulations and informed consent was obtained from all subjects and/or their legal guardian(s) where necessary. The inclusion criterion was age, from 12 to 55 years. The exclusion criteria were: a cervical spine with congenital anomalies; advanced degenerative changes; previous surgery; previous injuries; age less than 12 years and greater than 55 years; and a family history of mixed race.

The source data were acquired in isotropic voxel formats of 512 × 512 × 512 on a 64-slice multidetector Optima General Electric (GE) computed tomography machine with slice thickness of 3.75 mm and intervals of 1.25 mm thick. The reconstructed 1.25 mm × 1.25 mm stacks were displayed and studied in multiplanar reformat (MPR) and volume rendered (VR) forms to achieve as much anatomical detail as possible on a GE dual monitor workstation.

Each cervical vertebrae image was assessed by a postgraduate-level anatomist, an experienced radiologist whose subspecialties are neurological/spine and musculoskeletal radiology, and an orthopaedic/spine surgeon who helped in this study group the vertebrae properly into the bifid and non-bifid groups. The data were analyzed using the spreadsheet application Microsoft Excel version 15.0, and an appropriate graph was plotted.

### Determination of bifidity of the cervical vertebral spine

In this study, the spinous processes were categorized as either bifid or non-bifid, which is a modification of the classification system proposed by Duray et al. in 1999. Duray et al. classified the spinous processes as bifid, partially bifid, or non-bifid based on the presence of a clear cleft that results in two elongated projections in the bifid spinous process. Additionally, in order to be classified as bifid, the spinous process must have a bifurcation that separates both the tubercles and a portion of the spinous process itself. On the other hand, in the case of a partially bifid spinous process, there are two distinct tubercles present at the end of the spinous process. The spinous process is not divided into two branches and does not have a gap. The non-bifid spinous process has a rounded or flattened termination. While a median groove may be observed, the presence of two separate tubercles is absent.

**Figure 1:**
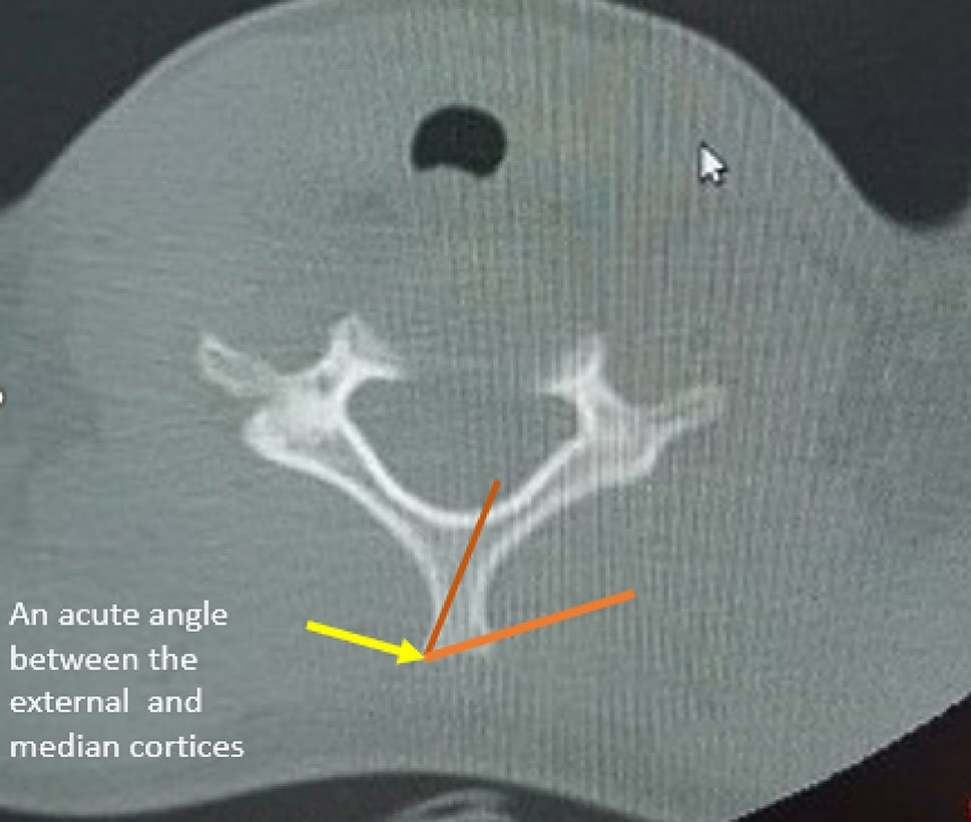
3D pictures of bifidity of C7 demonstrating criteria for classification to bifidity.

The classification of bifid and non-bifid in this study is determined by the existence of an acute angle between the outside (lateral) cortex of the spinous process and the middle cortex that separates the two outer cortices, as shown in Fig. 1.

### Ethical approval

All of the procedures were approved by the Ethical Committee of Anatomy Department, Ekiti State University Ado Ekiti, Ekiti State with protocol number ERCANA/2023/02/001 and were performed in accordance with the 1964 Helsinki declaration and its later amendments or comparable ethical standards.

## Results

**Figure 2:**
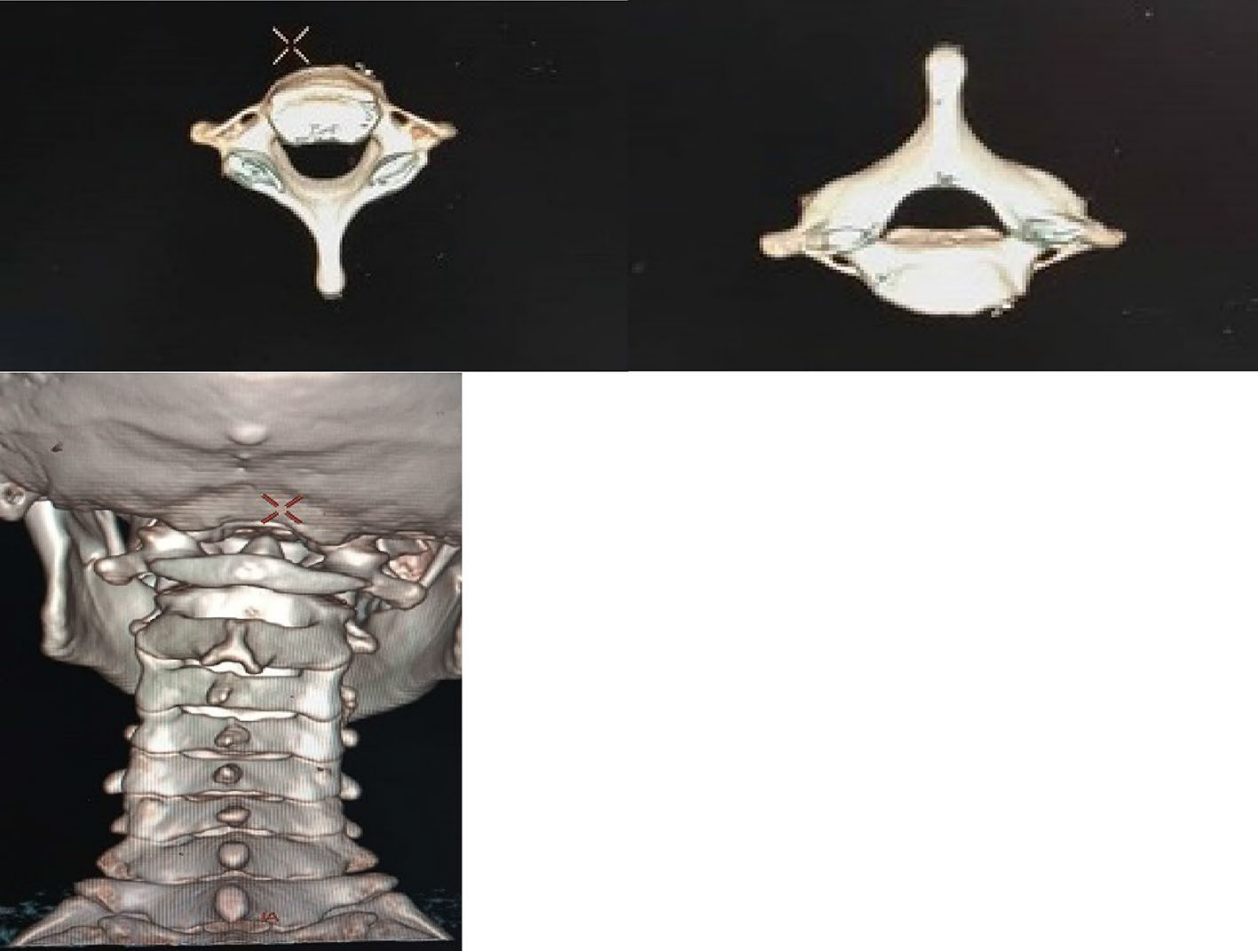
3D pictures of monofid C7.

**Figure 3:**
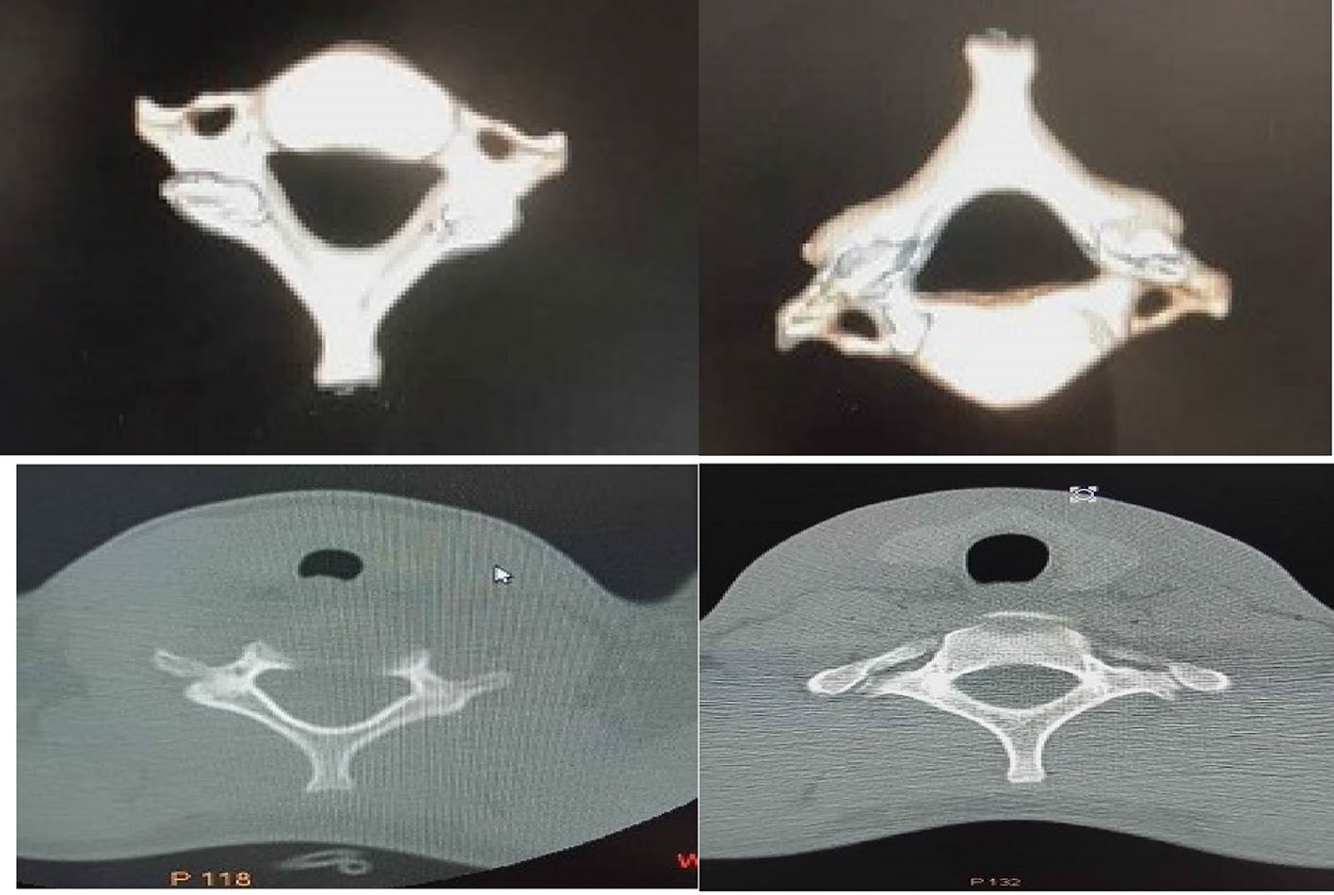
3D picture of bifid C7.

**Figure 4:**
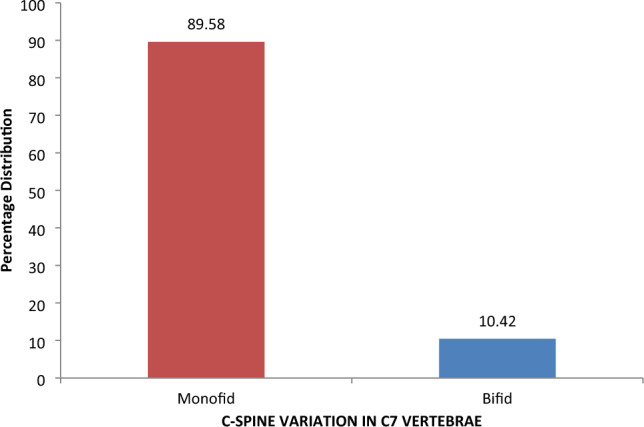
The percentage distribution of bifidity variation in C7 spine in Southwest Nigeria.

**Figure 5:**
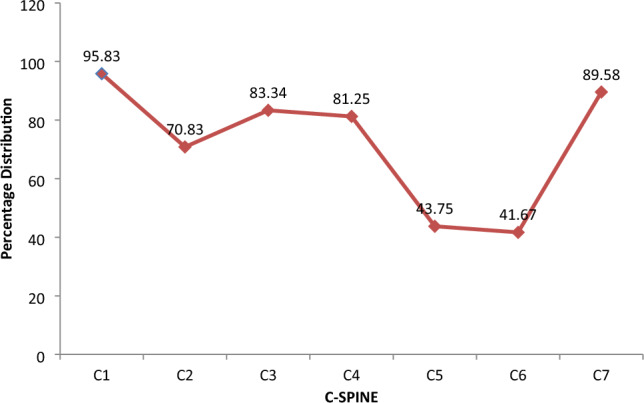
Comparison of the percentage incidence of monofidity in Cervical spine in Southwest Nigeria.

**Figure 6:**
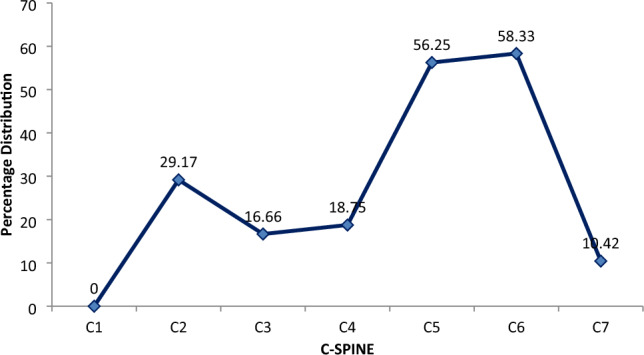
Comparison of the percentage incidence of bifidity in Cervical spine.

In this study, exclusion criteria were strictly followed, and due to poor record keeping, a 3D-rendered CT scan of 48 patients was harvested and analyzed. For ease of classification, subjects were classified as either bifid or monofid based on the presence of 4 or 2 bony cortices, respectively. It is also subjected to the agreement of two of the three observers, who are a radiologist, an anatomist, and an orthopaedic surgeon involved in the study. The Fig. 2 below represents the 3D pictures of monofid C7 while Fig. 3 represent 3D picture of bifid C7. Out of the 48 patients CT examined, five were found to be bifid according to criteria set by us; this stands at about 10.42% of the subject (Figs. 4 and 5). When compared to other cervical vertebrae, the incidence of bifidity is non-existent in the first cervical vertebra and highest in the C-6 vertebra (Fig. 6).

## Discussion

In medicine, the 7th cervical spinous process’ anatomical characteristics are frequently used to identify persons in forensic science It has been demonstrated that in humans, the interspinal and semispinalis cervicus muscles must grow in order for the process tip of the majority of cervical vertebrae to split into two^14^.

Only 0.3% of the subjects in Cho et al. large study from 2012 had C7 bifidity, but he divided the vertebrae into three categories: “bifid,” “somewhat bifid,” and “obviously monofid”^11^. This result is from the general population, and the stark contrast to our study, where 10.4% of the C7 are bifid, may also be the result of the rigid split into two groups and the racial element. The fact that this study’s vertebrae were produced in 3D, which is closer to the genuine anatomical morphology compared to Cho et al. 2012, 2D image assessment, suggests that methodology may have played a role. These findings contradicts the observation that White cervical vertebra generally exhibit higher bifidity^6^. In order to avoid complications from an incorrectly placed cervical catheter during anesthesia, appropriate cross inspection is preferable due to racial differences and the significant risk of choosing C7 over C6, which is mostly bifid.

Though counting down to locate lower vertebrae could be challenging, and assuming the last bifid vertebra could be misleading, especially in patients or people with short necks, C2 can be used as a landmark. This will necessitate the use of a fluoroscope or at least a simple x-ray for confirmation^11^. No bifidity was found in the 94 individual participants in Shore’s early twentith-century anatomical research among various South African races^2^. Moro et al. performed anatomical studies as well, though with fewer subjects, and saw just one case of 7th cervical spinal bifidity out of the 50 specimens examined; however, this study observed a comparably high percentage of bifidity^14^.

In spine surgery, the first and/or last rib-bearing vertebrae are often used as a reference point to count and navigate between vertebrae. As a result, unconventional segmentation can lead to errors in identifying surgical levels. The issue of failed instrumentation occurred, as reported^18^. When the first rib-bearing vertebra was used to count levels intraoperatively, as the T1 vertebra was mistakenly labeled rather than the C7 vertebra. Earlier general population studies by Cho et al. (2012) that suggested a decreased incidence of bifidity in the C7 cervical vertebra were contrasted with this study. When assuming monofidity of the C7 cervical is made, the clinician should be cautious of an even greater risk of incorrect instrumentation in the south-west Nigerian population. Since it may be used for forensic purposes in humans, this study will need to be further evaluated using a larger population, as well as similar research in other races. The goal of this study is to reconsider the use of monofidity of C7 as the baseline for cervical spine numbering across racial groups.

## Conclusion

Adequate knowledge of C7 morphology and incidence of bifidity is necessary for the spinal surgeon in order to avoid damage to the vertebral arteries, spinal cord, or nerve roots during fixation interventions involving the posterior cervical spine. The cervicothoracic junction is a challenging anatomical transition in spine surgery. In our research, we came to the conclusion that, based on this high level of bifidity in the population studied, other reliable investigative modalities should be undertaken to ascertain the vertebra in cases of orthopaedic and other medical procedures.

## Data Availability

The datasets used and/or analysed during the current study available from the corresponding author on reasonable request.

## Abbreviations

C2: Second cervical vertebra
C3: Third cervical vertebra
C4: Fourth cervical vertebra
C5: Fifth cervical vertebra
C6: Sixh cervical vertebra
C7: Seventh cervical vertebra
3D CT: Three dimension computed tomography
PACS: Picture archiving communication system
